# Postulations for the Migration Behavior of Amino Acids as Cations in Capillary Zone Electrophoresis

**DOI:** 10.1002/elps.202400205

**Published:** 2025-04-09

**Authors:** Peter Gross, Tom Huber, Isabel Lunow, Dominik Burkhard, Holger Seelert, Rolf Müller

**Affiliations:** ^1^ Hochschule Kaiserslautern, Campus Pirmasens Kaiserslautern Germany; ^2^ Helmholtz‐Institut für Pharmazeutische Forschung Saarland (HIPS), Campus E8.1 Saarbrücken Germany

**Keywords:** amino acids, capillary electrophoresis, conformation, hydrodynamic radius, migration behavior

## Abstract

Amino acids (AAs) in their cationic form at pH 2.2 and usual ionic strength show a non‐intuitive migration order in CZE. This is explained by setting up four postulates. The central points in these postulates are the influence of the AA side chain on the $pK_{a}$ value and the adoption of a defined, preferred conformation to build up the different $pK_{a}$ values. This conformation then also influences the hydrodynamic radius. The rotational orientation of an AA in the electric field aligns it, which also affects the hydrodynamic radius. Overall a special electrophoretical hydrodynamic radius is postulated and distinguished from the hydrodynamic radius, which is determined by the translational diffusion constant. With the help of the four postulates, the migration order could be explained. Glutamic acid has a special feature in this study: due to its observed higher mobility than the smaller and even higher charged aspartic acid, the hypothesis is that it would deprotonate first at the C5 and not at the C1 carboxylic group as all other AAs. This has the consequence of a more streamlined conformation and by that a faster migration in capillary electrophoresis.

**Abbreviations**
S‐CHANGE: side‐chain neighbor group effect

## 1 Introduction

Capillary electrophoresis (CE) in combination with UV/Vis‐absorption/conductivity detection or mass spectrometry with prior electrospray ionization (CE‐ESI‐MS) is a powerful method for characterization and analysis of biomolecules and also for determination of their physicochemical parameters, such as effective and limiting electrophoretic mobilities, etc. [1, 2, 3, 4, 5, 6, 7]. CE‐ESI‐MS works preferentially under acidic conditions with the amino acids (AAs) in their cationic forms. We started to work with CE‐ESI‐MS and found an unexpected migration sequence of AAs. Our aim is to explain this and propose a hypothesis for the migration behavior of AAs in the cationic form in the electric field.

**FIGURE 1:**
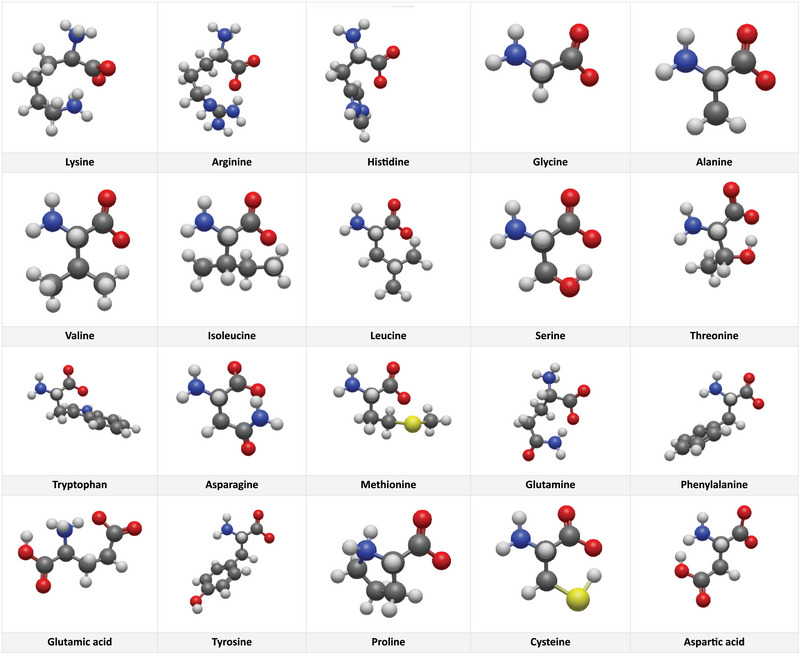
Visualized molecular models for the 20 proteinogenic AAs as cations at pH 2.2, according to postulate 1 and postulate 2 without further force‐field optimizations. Postulate 3 is then a consequence of the former postulates. The cathode is on the left, and the anode is on the right side for each molecule; accordingly, the amino group is on the left and the carboxy group is on the right as the upper part of the molecule.

The mobility of an ion in CE depends on (i) its charge, (ii) its hydrodynamic or Stokes radius (including potentially bound water molecules), (iii) its geometry, (iv) the temperature, (v) the viscosity, and (vi) the ionic strength of the surrounding solution. In the case of electrophoresis, the rotational orientation of the molecule in the electric field also plays a role as postulated herein. The separation behavior at a fixed pH of 2.2, as in this study, depends very strongly on the $pK_{a}$ value of the carboxylic acid. According to Vcelakova et al. [8], the $pK_{a}$ values of the 20 proteinogenic AAs fall in the range between 1.70 and 2.33. For the purpose of studying the migration behavior of the cationic forms of the AAs, the carboxylic $pK_{a}$ value implies two different pieces of information: (i) from the value a prediction of the charge of the cation is possible and (ii) they hint to the preferred conformation of the cationic form of the AA and by that to its hydrodynamic radius. We try to explain this by different Side‐CHAin‐Neighbor‐Group‐Effects (“S‐CHANGE”) in the AAs. Overall, four postulates were setup:

1. The S‐CHANGE influences the acidity of the carboxylic acid and forces an AA side chain into a preferred conformation to maximize its interaction with the positively charged amino group (see Table 1 for the different types of interactions).
2. All AAs experience a rotational orientation in the electric field. From a thermodynamic point of view, this could hypothetically lead to a complete loss of two rotational degrees of freedom (*y*‐, *z*‐axis) in the electric field (in the *x*‐axis) for the molecule.
3. Due to the chemical structure of an α‐AA postulate 2 leads sterically to an exposition of the C3 atom in a nonstreamlined form for all nonbasic AAs. The atoms in the side chain from the C4 position are more flexible and could adopt a streamlined form if the S‐CHANGE allows it (see, e.g., alanine in the top‐right of Figure 1).
4. The AAs adopt in consequence a specific electrophoretic hydrodynamic radius, which may differ from the translational diffusional hydrodynamic radius because of
  (a) the distinct frontal surface of the molecule towards the direction of movement, which is determined by both the S‐CHANGE (postulate 1) and the rotative alignment (postulate 2),
  (b) the bound water molecules, which build up an electrophoretical retardation effect according to the Debye–Hückel–Onsager theory [4, 9],
  (c) the loss of thermodynamic degrees of freedom. A maximum of four degrees of freedom is possible: two rotational and two translational degrees of freedom.

**TABLE 1:** Summary of the hypothesized S‐CHANGE for the AAs to explain the measured first $pK_{a}$ values of Vcelakova et al. [8].

No.	AA	$pK_{a}$ [8]	AA char.	$z_{\mathrm{sc}}$	*z*	S‐CHANGE
1	Lys	1.70 ± 0.07	Basic	1.00	1.24	CF: amino→C1
2	Arg	1.78 ± 0.07	Basic	1.00	1.28	CF: guanidino→C1
3	Cys	1.94 ± 0.03	Polar	0.00	0.35	Protonation: thiol→C1
4	Pro	1.96 ± 0.03	Aliphatic	0.00	0.37	HS: secondary amine
5	His	2.00 ± 0.08	Basic	1.00	1.39	CF: imidazole→C1
6	Asn	2.12 ± 0.02	Polar	0.00	0.45	HB: amide→C1
7	Glu	2.15 ± 0.02	Acidic	−0.01	0.46	CF: amino→C5‐carboxylate
7	Thr	2.15 ± 0.02	Polar	0.00	0.47	HB: alcohol→C1
8	Gln	2.16 ± 0.02	polar	0.00	0.48	HB: amide→C1
9	Met	2.17 ± 0.02	Aliphatic	0.00	0.48	HS: thioether→amine
9	Ser	2.17 ± 0.02	Polar	0.00	0.48	HB: alcohol→C1
10	Phe	2.18 ± 0.02	Aromatic	0.00	0.49	HS: cationic‐π‐aromatic interaction
11	Tyr	2.22 ± 0.01	Aromatic	0.00	0.51	HS: cationic‐π‐aromatic interaction
12	Val	2.26 ± 0.02	Aliphatic	0.00	0.53	HS: isopropyl→amino
13	Asp	2.28 ± 0.03	Acidic	−0.03	0.52	HB or inductive electron drawing
14	Ile	2.30 ± 0.01	Aliphatic	0.00	0.56	HS: sec‐butyl→amino
15	Leu	2.31 ± 0.02	Aliphatic	0.00	0.56	HS: iso‐butyl→amino
16	Gly	2.32 ± 0.01	Aliphatic	0.00	0.57	No effect
16	Trp	2.32 ± 0.01	Aromatic	0.00	0.57	No effect
17	Ala	2.33 ± 0.01	Aliphatic	0.00	0.57	No effect

Abbreviations: AA char., AA character; CF, Coulombic force; HB, hydrogen bonding; HS, hydrophobic shielding against hydration and by that a smaller extent of charge dissipation; *z*, charge number; $z_{\mathrm{sc}}$, charge number of the side chain.

This paper attempts to verify these postulates by analyzing the 20 proteinogenic AAs with the following methodology: We attempt to explain the observed $pK_{a}$ values with the S‐CHANGE model and have decided to use only the high‐quality $pK_{a}$ data from Vcelakova et al. [8]. Based on this explanation, an attempt is made to visualize the AA conformation by creating simple three‐dimensional models without further force field optimizations. All the AAs are aligned rotationally with respect to the tackling electrical forces. The actual effective electrophoretic mobility of each AA is measured by CE, and from these data an apparent electrophoretic hydrodynamic radius $R_{e}^{\prime }$ is calculated. The word “apparent” in this expression refers to the fact that the actual effective mobility is used and not the limiting mobility of the ions at infinite dilution, which would be the more accurate parameter in this context. Nevertheless, this value is compared with the hydrodynamic radius $R_{0}$, determined via the translational diffusion constant. Furthermore, a frontal surface radius $R_{f}$ is measured in the modeled molecule, and this is also compared with $R_{0}$ and the measured actual effective mobility.

## 2 Materials and Methods

### 2.1 Materials

Tryptophan and tyrosine were sourced from SERVA GmbH, asparagine from Thermo Fisher GmbH, arginine from Alfa Aesar, histidine from Merck KGaA, and all other AAs were procured from Carl Roth with a purity of 98.5 % or higher. Hydrochloric acid, sodium hydroxide, and phosphoric acid were sourced from Roth, sodium dihydrogen phosphate was procured from Merck KGaA, and the acetone was obtained from VWR Chemicals. The concentration in the specified run is 0.5 mg/mL for all AAs, with the exception of 1.0 mg/mL for Gly and 1.5 mg/mL for Phe. The AAs were dissolved in the background electrolyte (BGE) with 2 % (v/v) acetone as an electroosmotic flow (EOF) marker. A peak assignment is based on single AA runs in combination with Phe as a mobility marker (not shown here).

### 2.2 Capillary Electrophoresis

As BGE 50 mM ${\mathrm{NaH}}_{2}{\mathrm{PO}}_{4}$ is pH‐adjusted to pH 2.2 with the aid of 85 % (w/w) phosphoric acid, it has a conductivity of 5.67 mS/cm and an ionic strength of 50 mM. A CE instrument PA 800plus from Beckman Coulter is operated with the software 32 Karat version 9.1 at a separation voltage of 20 kV with normal polarity at 25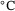 and a current of 89 μA. A fused silica capillary (Polymicro Technologies) with 75 μm id (effective length = 0.5 m; total capillary length = 0.6 m) and 300 μm od was used for separation. The preconditioning of the capillary was done by rinsing 40 min with BGE. For each run, the capillary was rinsed with 0.1 M HCl (2 min), distilled water (1 min), 0.1 M NaOH (2 min), and finally with BGE (5 min). Due to the observed peak asymmetries in many cases, the CE run is evaluated via a Haarhoff‐Van‐der‐Linde function by using Peakfit (Systat Software GmbH). From this, the correct migration time ($t_{m}$ value) of the undisturbed migration can be calculated, which would correspond to an ideal Gaussian profile [10]. The graphs of the CE runs and the evaluations were created using Origin Pro17G (OriginLab Corporation) or the statistic software RStudio [11].

### 2.3 Calculation of AA Charges

The formation of the negative decadic logarithm to the Henderson–Hasselbalch equation (Equation 1) results in its reshaped form (Equation 2).

(1) $$\begin{array}{c} \text{pH}=pK_{a}+\text{lg}\frac{c_{A^{-}}}{c_{\text{HA}}}, \end{array}$$

(2) $$\begin{array}{c} \Leftrightarrow \frac{c_{A^{-}}}{c_{\text{HA}}}=10^{\text{pH}-pK_{a}}. \end{array}$$

At pH 2.2, the amino group of each AA is completely protonated, the charge number *z* of a single AA can thus be calculated from Equation (4), where $z_{\mathrm{sc}}$ stands for side chain's charge number, which can also be clearly assigned by this equation at pH 2.2. The individual *z*‐values for the AAs are listed in Table 1.

(3) $$\begin{array}{ccc} z & = & z_{{\text{NH}}_{3}^{+}}+z_{\mathrm{sc}}-\frac{c_{A^{-}}}{c_{A^{-}}+c_{\text{HA}}}=1+z_{\mathrm{sc}}-\frac{c_{A^{-}}}{c_{\text{HA}}\left(\frac{c_{A^{-}}}{c_{\text{HA}}}+1\right)}\\ & = & 1+z_{\mathrm{sc}}-\frac{\frac{c_{A^{-}}}{c_{\text{HA}}}}{\left(\frac{c_{A^{-}}}{c_{\text{HA}}}+1\right)}, \end{array}$$

(4) $$\begin{array}{cc} & \Leftrightarrow z=1+z_{\mathrm{sc}}-\frac{10^{\text{pH}-pK_{a}}}{\left(10^{\text{pH}-pK_{a}}+1\right)}. \end{array}$$

### 2.4 Calculation of the Electrophoretical Mobility

With a total capillary length $L_{t}$ of 0.6 m and an applied voltage U of 20 kV, an electrical field strength of 33 333.33 V/m results during separation. The diode array detector is located at the detection window with an effective capillary length $L_{e}$ of 0.5 m. The apparent electrophoretic mobility $\mu_{\text{app}}$, based on the migration time $t_{m}$, could be calculated directly using the following Equation (5).

(5) $$\begin{array}{c} \mu_{\text{app}}=\frac{L_{e}\cdot L_{t}}{t_{m}\cdot U}. \end{array}$$

The calculation of the effective electrophoretic mobility μ is achieved by the use of acetone as an uncharged molecule (EOF marker). From the respective migration time $t_{\text{EOF}}$ of 50.24 min, the mobility $\mu_{\text{EOF}}$ is calculated according to Equation (5). The relationship between μ, $\mu_{\text{app}}$, and $\mu_{\text{EOF}}$ is shown in Equation (6).

(6) $$\begin{array}{cc} \mu & =\mu_{\text{app}}-\mu_{\text{EOF}}. \end{array}$$

### 2.5 Calculation of Electrophoretical Hydrodynamic Radius

A hydrodynamic radius is derived from the equilibrium condition of the electrophoretic migration and is herein called apparent electrophoretical hydrodynamic radius $R_{e}^{\prime }$. The velocity v of the analyte is calculated by Equation (7). Together with the calculated charge number *z* and the elementary charge *e*, $R_{e}^{\prime }$ is then calculated with the help of Equation (8). It should be mentioned that Equation (8) is a simplified form of the equation for the calculation of the Stokes radius from the electrophoretic mobility. In a more precise form of this equation, the limiting ionic mobility of the analyte should be used instead of the actual effective mobility [6, 7].

(7) v=μ·E.

(8) $$z\cdot e\cdot E=6\pi \cdot R_{e}^{\prime }\cdot \eta \cdot v\Leftrightarrow R_{e}^{\prime }=\frac{z\cdot e\cdot E}{6\pi \cdot v\cdot \eta }=\frac{z\cdot e}{6\pi \cdot \eta \cdot \mu }.$$

In the following calculations for *e*, a value of $1.602176634\times \;10^{-19}$ C and for the viscosity η a value of 0.891 mPa·s is used.

### 2.6 Molecular Modeling

Molecular modeling is performed with the open‐source software Avogadro [12] with respect to the expected S‐CHANGE at pH 2.2. The groups are rotated so that neighboring groups approach each other due to effects such as hydrogen bonding or electrostatic interactions etc. (see Table 1). The preferred conformers are then aligned in the electrical field, the positively charged amino group towards the cathode and the negatively charged carboxyl group towards the anode. Charged side chains are also aligned. The radius of the frontal surface $R_{f}$ is determined by measuring half the distance between the amino group and the atom furthest below it using the Avogadro software.

## 3 Results and Discussion

### 3.1 Postulate 1: AA Charge and Conformation at pH 2.2

The $pK_{a}$ values of Vcelakova et al. [8] are summarized and sorted in Table 1, together with their respective charge numbers. The values and order of the $pK_{a}$ values of the most acidic AAs, aspartic and glutamic acids, and of the two basic AAs, lysine and arginine, are in agreement with the expected values and order but the $pK_{a}$ of the third basic AA, histidine, is higher than expected and it occupies only position 5. Position 3 goes to the polar uncharged AA cysteine and position 4 to the aliphatic AA proline with its secondary amine. In Table 1, hypothesized explanations for the observed S‐CHANGEs are given. On the basis of the observed S‐CHANGEs and postulate 1, proposals of expected conformations for the individual AAs are visualized and shown in Figure 1.

### 3.2 Postulates 2 and 3: Rotatory Alignment of the AAs in the E‐Field and Protruding of the C3 Atom

In Figure 1, the AAs are depicted in their preferred conformations according to our supposed S‐CHANGE model without further force–field optimizations, together with their rotatory alignment in the electrical field. AAs with an uncharged side chain, exposed in the electrical field, are aligned as shown in Figure 1 (see, e.g., alanine). The electrical force orientates the amino‐ and the acid group on a theoretically horizontal line opposed to each other with the C3 atom exposed perpendicular to this in a strict spatially fixed position. Overall, this orientation is not streamlined at all due to the protruding C3 atom, but the following positions could be arranged in a more favorable way (see, e.g., methionine in Figure 1). The rotational orientation of AAs with a third charge in the side chain is estimated by the following procedure: The three electrical forces, in dependency on the individual charges, provoke by that a torque and orientate the molecules in the form of a triangle in the electrical field. The two charges with the same sign build the base of the triangle towards one electrode, and the third charge is the apex towards the opposite electrode. Lysine in Figure 1 is an example of that.

### 3.3 Postulate 4: Apparent Electrophoretical Hydrodynamic Radius

**FIGURE 2:**
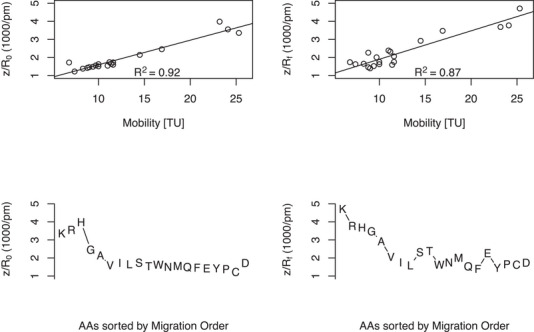
Upper part: The quotients $z/R_{x}$ for the different radii ($R_{f}$: frontal surface radius; $R_{0}$: Stokes radius) is drawn against the mobility in Tiselius units ($\mathrm{TU}=1\times 10^{-9}\;m^{2}\;V^{-1}\;s^{-1}$), together with a linear regression. Lower part: The same $z/R_{x}$ values are drawn against the real observed migration order in the electrophoresis experiment.

In Table 2, the relevant physicochemical data for the AAs are summarized. The data are either calculated on the basis of experimentally determined values (mobility μ, apparent electrophoretical Stokes radius $R_{e}^{\prime }$), are modeled (frontal surface radius $R_{f}$), or taken from the literature (diffusionally determined Stokes radius $R_{0}$). By comparison of $R_{0}$ [13] versus the herein‐determined $R_{e}^{\prime }$ radius, it becomes obvious that the latter is significantly larger. Even one has to keep in mind that the $R_{0}$ values are measured at pH 3.5, which means a more deprotonated carboxylate group. From this point of view, one would expect that the degree of hydration would be higher than at pH 2.2 and consequently also the Stokes radius. Regarding postulate 3, this could be at least hypothetically explained by the unfavorable protruding of C3 and perhaps also by using the actual mobility instead of the limiting ionic mobility. In the upper part of Figure 2, the different quotients $z/R_{x}$ are drawn against the mobilities together with a regression line for each. The plot of $z/R_{0}$ versus μ fits with an $R^{2}$ value of 0.92, which is slightly better than, what we observe for plotting $R_{f}$ versus μ with a value of 0.87. The purely statistical analysis obscures important physical details; therefore, a closer look is required.

**TABLE 2:** Summary of the relevant physicochemical parameters for the 20 proteinogenic AAs ($t_{m}$, migration time; μ, electrophoretic mobility; $R_{e}^{\prime }$, experimentally determined apparent electrophoretical Stokes radius; $R_{f}$, modeled radius of the frontal surface during electrophoretic migration; and $R_{0}$, the literature value for the diffusional Stokes radius [13]).

		$t_{m}$	μ	$R_{e}^{\prime }$	$R_{f}$	$R_{0}$	*z*	*z*/$R_{0}$	*z*/$R_{f}$
No.	AA	(min)	$\left(\frac{10^{-9}m^{2}}{\text{Vs}}\right)$	(pm)	(pm)	(pm)		($\frac{10^{3}}{\text{pm}}$)	($\frac{10^{3}}{\text{pm}}$)
1	Lys	8.25	25.33	467	263	369	1.24	3.36	4.72
2	Arg	8.59	24.12	506	339	360	1.28	3.56	3.78
3	Cys	20.17	7.42	450	215	286	0.35	1.22	1.63
4	Pro	18.83	8.30	425	224	268	0.37	1.38	1.65
5	His	8.87	23.20	572	377	349	1.39	3.98	3.69
6	Asn	16.69	10.00	429	257	298	0.45	1.51	1.75
7	Glu	18.21	8.75	502	203	314	0.46	1.42	2.27
7	Thr	15.63	11.01	407	197	304	0.47	1.55	2.39
8	Gln	17.39	9.40	487	315	323	0.48	1.49	1.52
9	Met	17.08	9.66	474	239	308	0.48	1.56	2.01
9	Ser	15.45	11.20	409	207	276	0.48	1.74	2.32
10	Phe	17.82	9.05	517	195	335	0.49	1.46	2.51
11	Tyr	18.21	8.75	556	195	357	0.51	1.43	2.62
12	Val	15.10	11.58	437	257	332	0.53	1.60	2.06
13	Asp	21.20	6.81	728	299	302	0.52	1.72	1.74
14	Ile	15.10	11.58	461	228	324	0.56	1.73	2.46
15	Leu	15.22	11.45	467	297	339	0.56	1.65	1.89
16	Gly	11.42	16.91	322	164	232	0.57	2.46	3.48
16	Trp	16.69	10.00	544	176	350	0.57	1.63	3.24
17	Ala	12.84	14.48	376	195	266	0.57	2.14	2.92

#### 3.3.1 Glutamic/Aspartic Acid Dilemma

**FIGURE 3:**
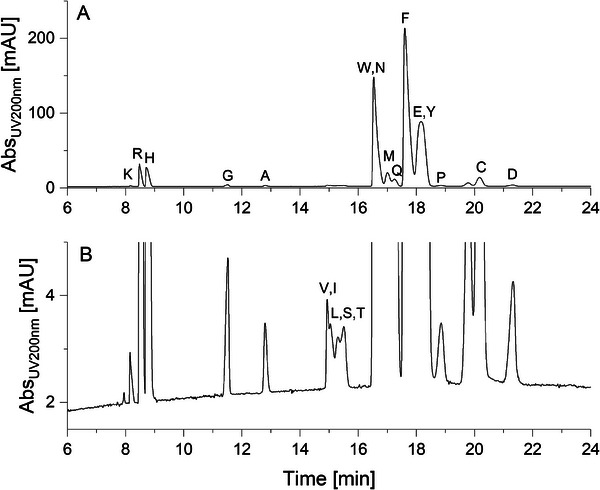
A: Electropherogram of the 20 proteinogenic AAs with single letter peak labeling. The unassigned peak before C is cystin, which is not further evaluated. B: Enlarged view of A. (Concentration is 0.5 mg/mL for all AAs, with the exception of 1.0 mg/mL for Gly and 1.5 mg/mL for Phe; pH = 2.21; 20 kV separation voltage, current 89 μA, conductivity = 5.67 mS/cm; effective length = 0.5 m; total capillary length = 0.6 m; capillary diameter = 75 μm; normal polarity; *T* = 298 K.)

In the lower part of Figure 2, the same *y*‐values (as in the upper part) are taken and plotted against the migration order as it was observed in the experiment in Figure 3. In the case of $z/R_{0}$, the middle part is quite similar to the real observed migration order but has deviations for aspartate and does not place it in the right order compared to glutamate. In this work and Hirayama et al. [1], glutamic acid is faster than aspartic acid, even though its calculated charge number is smaller (−0.06). As we see in Table 2, the $R_{e}^{\prime }$ value of aspartate lies extremely far away from its diffusional correlate and also obviously far away from all other AAs. The difference between aspartate and glutamate with a $R_{e}^{\prime }$ of 728 pm versus 502 pm is surprising in view of the additional methylene group in glutamate. The hypothetical assumption that the C5 would be more acidic than the C1 carboxylic acid in glutamate, as was already done in Table 1, provides an explanation. The S‐CHANGE between the C5 carboxylic acid and the amino group leads to a very streamlined conformation of glutamate versus aspartate (see Figure 1). This is confirmed by the data shown in Table 2, with $R_{f}$ values of 203 pm for glutamate versus 299 pm for aspartate. Another explanation would be that E in our experiment is in a different hydrogen bonding environment or is less disturbed by effects based on the Debye–Hückel–Onsager theory than D. The study by Zuskova et al. [4] supports the last assumption because in their experimental setup D was faster than E.

#### 3.3.2 Basic AAs dilemma

In the case of $z/R_{0}$, a reverse migration sequence for the basic amino acids (H, R, K) in the upper part of the mobility order is observed. The use of $z/R_{f}$ eliminates these problems with the basic amino acids and puts them in the correct sequence (see Figure 2, lower right). It can therefore be assumed that postulate 2 applies particularly to basic amino acids as these have a higher charge number *z* and therefore a stronger electrical force. In this sense, it is conceivable that postulate 2 has a graduated validity and depends on the balance of forces between thermodynamics (degrees of freedom for movements) and electrostatics.

### 3.4 Interpretation of the Electropherogram on the Basis of the Four Postulates

In Figure 3, the measured electropherogram for the 20 proteinogenic AAs is depicted. The different peak heights are mainly due to the different molar extinction coefficients of the individual AAs, their concentrations are largely comparable. The basic AAs dilemma was already described in Section 3.3.2. Glycine possesses the standard charge number of *z* = 0.57 (see Table 1) for the aliphatic AAs, but it is the smallest AA and therefore the fastest in the group of the AAs with standard charge. The modeled $R_{f}$ value of only 164 pm is in accordance with practice and theory and has, as expected, the smallest value for all AAs. The introduction of one methyl group in glycine leads to alanine, for which the migration time increases by 1.42 min. This value is threefold higher compared to the migration time increase of only 0.12 min going from valine to leucine, which differs by one methylene group. This hints towards postulate 3 because the additional methylene group in leucine is positioned beyond the C3 position. Ile with a sec‐butyl group is a little bit faster than leucine due to the smaller $R_{f}$ value of 228 pm versus 297 pm with the same charge as leucine. Serine migrates, as expected, faster than threonine with an additional methyl group. Tryptophan possesses a charge number of *z* = 0.57 and migrates for that reason faster than the smaller phenylalanine with a charge number of only *z* = 0.49. Asparagine comigrates with tryptophan, it is smaller but the charge number of *z* = 0.45 is also smaller. In spite of the fact that methionine has two additional alkylic C atoms, compared to cysteine, it migrates faster with its higher charge number of 0.48 versus 0.35 for cysteine. Glutamine possesses also a charge number of *z* = 0.48 but has a larger $R_{f}$ value of 315 pm versus 239 pm for methionine, probably because of its unfavorable conformation as a result of the interaction between the side chain amide and the C1 carboxylic group (see Table 1). Phenylalanine possesses a similar charge number 0.49, but it is bigger. The glutamic/aspartic acid dilemma was already described and discussed in Section 3.3.1. Tyrosine has a charge number of *z* = 0.51, which is slightly higher than the charge of phenylalanine but has simultaneously a higher molecular mass and the ability to arrange hydrogen bonds with water. Proline has the second smallest charge number of only *z* = 0.37, which explains its low mobility. Cysteine carries the smallest charge of all AAs at pH 2.2, probably because of its acidic hydrogen atom, which leads to a surprisingly small $pK_{a}$ value. The slowest AA is aspartate with a charge number of *z* = 0.52, which is higher than the charge number of glutamate (0.46) or asparagine (0.45) (see also Section 3.3.1). The smaller mobility of glutamate in comparison to glutamine could be based on a stronger hydrogen bonding of the carboxylic acid to the surrounding water in comparison to the amide.

## 4 Concluding Remarks

Our analysis indicates a specific electrophoretic Stokes radius due to the orientation of the AAs in the electric field and the associated implications, for example, loss of two rotational degrees of freedom, etc. This particularly affected AAs with a basic side chain. The thermal movement of the molecules presumably works against this orientation. A practical consequence thereof would be that postulate 2 becomes less important with increasing temperature and this effect can possibly be used to optimize a CE separation. Our hypothesis suggested that, among all AAs, glutamate could be the only one, which deprotonates first in the side chain, not in the C1 carboxylic acid. An explanation for this result, in view of a possible degradation of glutamate, is unlikely because of the CE‐ESI‐MS results of Hirayama et al. [1]. They found the same migration order as here between glutamate and aspartate and measured the intact molecules per mass spectrometry in the acidic BGE. Another conceivable explanation for the fundamental difference between aspartate and glutamate could be a different hydrogen bonding pattern among the two carboxylic acids for example. Since this difference would be independent of the electrical field, this should be also found in the translational diffusional determined Stokes radii, but this is not the case (see Table 2). Therefore, if the hydrogen bonding pattern is the reason for the difference, then it must rely on the special electrophoretic retardation effect. This study could not answer if the H‐bonding pattern or the speculated different acidity is the reason for the observed differences between aspartate and glutamate.

Nevertheless, glutamate takes a special role among all AAs, concerning, for example, the ammonia metabolism, where the side chain is cyclically amidated and deamidated. In this amidation/deamidation cycle, the α‐amino group is perhaps catalytically activated by direct interaction with the side chain carboxylic acid. Also, glutamate is the precursor of proline with a heterocyclic five‐ring structure, built by connecting the side‐chain carboxylic acid with the α‐amino group. To what extent the hypothesized interaction between the side chain carboxylic and the amino group plays a role in these biochemical pathways could be only speculated here, and this has to be examined in further studies.

## Conflicts of Interest

The authors declare no conflicts of interest.

## Data Availability

The data that support the findings of this study are available from the corresponding author upon reasonable request.
